# Bioactive Glasses in Periodontal Regeneration: Existing Strategies and Future Prospects—A Literature Review

**DOI:** 10.3390/ma15062194

**Published:** 2022-03-16

**Authors:** Valeria Cannillo, Roberta Salvatori, Stefania Bergamini, Devis Bellucci, Carlo Bertoldi

**Affiliations:** 1 Department of Engineering “Enzo Ferrari”, University of Modena and Reggio Emilia, Via P. Vivarelli 10, 41125 Modena, Italy; devis.bellucci@unimore.it; 2 Department of Industrial Engineering and BIOtech Research Center, University of Trento, 38123 Trento, Italy; roberta.salvatori@unitn.it; 3 Department of Surgery, Medicine, Dentistry and Morphological Sciences with Transplant Surgery, Oncology and Regenerative Medicine Relevance (CHIMOMO), University of Modena and Reggio Emilia, 41124 Modena, Italy; stefania.bergamini@unimore.it (S.B.); carlo.bertoldi@unimore.it (C.B.)

**Keywords:** bioactive glasses, periodontal regeneration, bone regeneration, scaffolds, coatings, in vitro tests, in vivo tests, animal model, clinical trials

## Abstract

The present review deals with bioactive glasses (BGs), a class of biomaterials renowned for their osteoinductive and osteoconductive capabilities, and thus widely used in tissue engineering, i.e., for the repair and replacement of damaged or missing bone. In particular, the paper deals with applications in periodontal regeneration, with a special focus on in vitro, in vivo and clinical studies. The study reviewed eligible publications, identified on the basis of inclusion/exclusion criteria, over a ranged time of fifteen years (from 1 January 2006 to 31 March 2021). While there are many papers dealing with in vitro tests, only a few have reported in vivo (in animal) research, or even clinical trials. Regardless, BGs seem to be an adequate choice as grafts in periodontal regeneration.

## 1. Introduction

Bioactive glasses (BGs) were discovered by Larry L. Hench in 1969; the original composition, named Bioglass^®^ 45S5 (45S5 from here on) [1,2] displayed outstanding properties such as bone regeneration capabilities and antibacterial activity. In fact, such BG is an amorphous and biocompatible silica oxide-based inorganic material able to induce surface property responses resulting in the formation of a bond between the bone and the glass itself. From 45S5, a family of BGs was developed; such glasses are usually composed by oxides of Si, Ca, P, and Na. The release of these ions into the tissues could induce specific cell responses and explain their biologic properties [3]. In recent years, such compositions have been further modified to incorporate also the so-called therapeutic ions. Thus, strontium, magnesium, copper, silver, zinc, lithium have been successfully included in BG formulations, and the resulting BGs displayed favorable effects in terms of overall biological response [4,5,6,7,8,9,10]. BGs could regulate gene expression, protein synthesis, and cell-mediated mineralization [11]. Moreover, the incorporation of metallic ions into BGs induces angiogenesis [12].

Even if silica oxide-based glasses are the most used, other systems were also developed, such as phosphate-based BGs. These glasses are mainly based on P_2_O_5_, Na**_2_**O, and CaO and modifying oxides, for example CuO [13], ZnO [14], Ag_2_O [15], Fe_2_O_3_, TiO_2_ [16], and SrO [17], which improve their stability [18]. They have degradation times ranging from hours to years depending on the composition and can be used as a scaffold for tissue regeneration [19].

In general, the surface of BGs, when soaked in physiological fluids, undergoes a complex ion exchange mechanism with the medium, inducing the formation of precipitates and subsequently hydroxyapatite deposition. This mechanism could explain the effectiveness of these BGs to bind to bones and the wide number of studies on BGs as supporting materials for bone tissue engineering and tooth remineralization [20]. Moreover, this superficial layer plays an important role in favoring cells migration and adhesion. Thus, by the same mechanism of ion exchange, bioactive glasses have been shown to regulate gene expression and promote cells differentiation, two important steps in tissue regeneration and repair [21].

BGs can be produced by melt-quenching or sol-gel routes [22]. In the first method, the oxides (or their precursor such as nitrates or carbonates) are melted together and rapidly quenched to obtain a frit, which is subsequently ground and sieved. The sol-gel route requires a specific chemical approach with precursor polymerization at room temperature to form the glass network [23]. Even if the melt-quenching route is the most employed, sol-gel offers some advantages, i.e., the synthesis temperature is relatively low, the composition ratio can be easily modified, the standardization of the product’s particle size can be organized to obtain pure samples with high uniformity, and constituents are allowed to be doped [24].

BGs could be prepared with a different degree of porosity, increasing the surface area, providing different patterns of support for cell adhesion, cell migration, and modifying cellular response and growth [25]. Indeed, the porosity could act as a guide for angiogenesis and trophic supply [26].

BGs show a great versatility of composition and, consequently, of use. Up to date, BGs have been produced in bulk form, particles or nanoparticles, granules, scaffolds, and coatings (e.g., [27,28,29,30,31,32,33]). As far as the coatings are concerned, BG can be deposited on metallic substrates, such as prostheses or dental implants, to improve the biological behavior of the system; in this case the analysis of potential residual stresses due to the mismatch of coefficients of thermal expansion between the glass and the substrate should be included [34,35].

Moreover, BGs can be combined with other materials to obtain bioactive composites [36], or their porous structure could be loaded with bioactive molecules for a local release of active principles [37]. However, not all substances can be favorably mixed with BGs, e.g., polylactic acid undergoes a biodegradation producing an acid environment that neutralizes the alkaline environment generated by BGs, favoring bacteria proliferation [26,38]. On the contrary, a BG containing Zn and Mg was incorporated into alginate networks to improve mechanical, antibacterial, and biologic properties in dentistry [39]. The use of chitosan in dentistry [40,41,42] represents a novel choice. Chitosan is neutral after degradation, so the alkaline antibacterial environment proper of BG can carry out its function. This composite material has the potential to induce bone regeneration and could be significant for promoting the proliferation and metabolism of human periodontal ligament cells and the metabolism of human bone marrow stromal cells [38].

**Figure 1:**
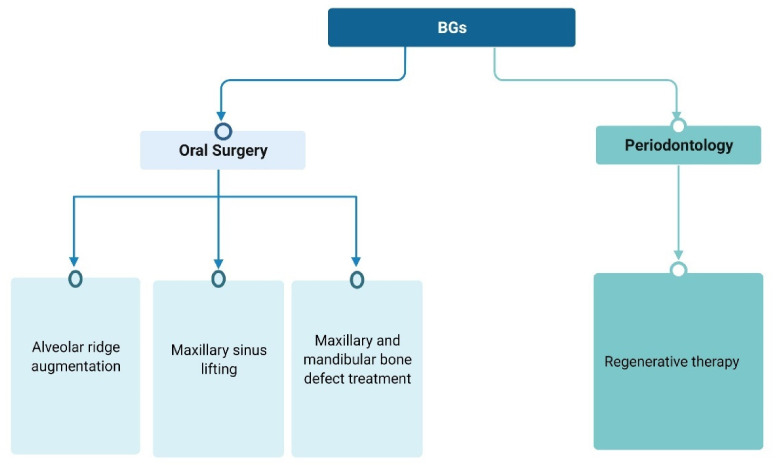
BGs use as regenerative surgical treatment in dentistry.

In tissue engineering, a major focus of attention is the recognition and activation of adult stem cells. Periodontal tissue harbors a great amount of different cell types. Inside the periodontal tissue, mesenchymal cells and adult stem cells have been identified and have been demonstrated to be able to differentiate in periodontal ligament specific fibroblasts [43,44,45]. The main role of BGs in dental and periodontal restoration and regeneration is the proper stimulation of those cells, to produce new tissues and to favor periodontal attachment. In fact, an ideal material for periodontal regeneration should be able to elicit and promote cell proliferation and differentiation and should have adequate mechanical properties, resembling the target tissue. Additionally, the material should be suitable for the different clinical features of the possible periodontal defects (as illustrated in Figure 1).

Moreover, BGs must show antibacterial or bacteriostatic properties and interesting interplays with the immune system.

BGs seem to hinder bacterial growth and development, and this task can be improved by loading them with antibiotics or doping with bactericidal ions (such as silver) to avoid the emergence of resistant strains [12]. Increasing evidence reveals the mechanisms and cellular pathways of interactions between BGs and the immune system. In particular, the influence of BGs on immune cells (e.g., macrophages, monocytes, DCs) has been demonstrated, as a key factor to regulate the immune response to these biomaterials. The interaction between BGs and the immune system (especially with macrophages and monocytes) appears to be bi-univocal and dynamic. The secretion of specific cytokines by immune cells seems to be significantly affected by the degradation kinetics and degradation products (mostly ions) of BGs [46]. In this context, it should be pointed out that immune cells can conversely influence the degradation process of BGs, which consequently affects the structure, morphology, mechanical properties, and ion release behavior of BGs. However, to develop feasible, effective, and advanced BG-based grafts or also biomedical devices with immunomodulatory capability for tissue regeneration, several major challenges remain and have to be addressed in future studies [47].

Periodontal diseases are oral infections characterized by gingival inflammation, clinical attachment loss, and alveolar bone resorption [48]. Oral microbiota represents one of the most effective risk factors for periodontitis and for periodontal regenerative therapy failure [49,50]. The current trend in periodontology is to preserve as much as possible the periodontal attachment and to regenerate the lost parts. Therefore, tissue engineering is a topical subject and an emerging field based on the combination of advanced surgical techniques, stem cells, growth factors, angiogenesis, and biomaterials.

**Figure 2:**
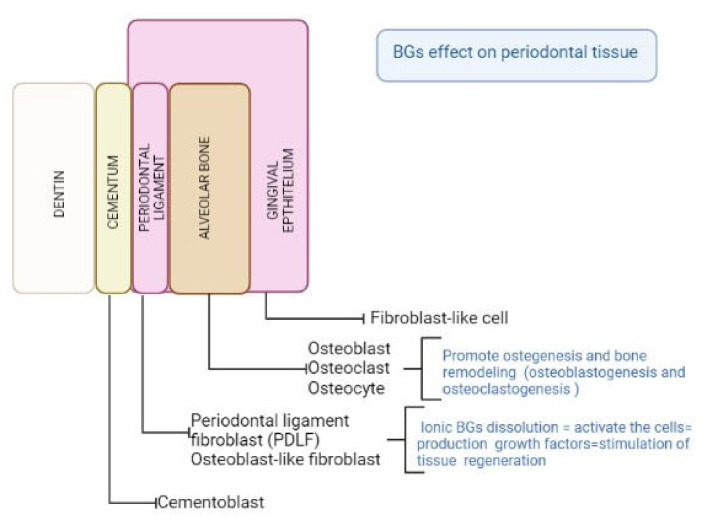
Schematic illustration of the BGs effect on periodontal cells.

Since BGs show intriguing properties considering their biocompatibility, cell response, adaptability to clinical features and antibacterial properties, they have been incorporated as filler in scaffolds for tooth and periodontal regeneration [24] (Figure 2).

Therefore, the aim of this review is to address the state of the art of the use of BGs in regenerative periodontal surgery, based on their biological behavior, by assessing any progress in the experimental in vitro and in vivo (animal model) tests and by evaluating clinical outcomes.

## 2. Materials and Methods

The present review was written following the criteria and guidelines for literature review [51].

### 2.1. Primary and Secondary Outcomes

The primary outcomes of interest in this review were the clinical changes of periodontal indices (e.g., pocket probing depth—PPD, recession depth—REC, clinical attachment level—CAL) achieved in patients who underwent regenerative periodontal surgery using BGs as graft.

The secondary outcomes (in vitro and in vivo outcomes) of interest were:

1.  The changes in human periodontal ligament cells (hPDLCs), human periodontal ligament stem/progenitor cells (HPDLC), and human bone marrow stromal cells (hBMSC) as cell viability, cell proliferation, cell differentiation, enhanced mineralized tissue formation.
2.  The changes in fibroblast/osteocyte cell lines (RAT primary osteoblastic cells—RPO; pre-osteoblasts murine cells—MC3T3-E1; human osteosarcoma cells—MG63; odontoblast-like cells—MDPC-23; oral mucosa fibroblasts cells—MM1; osteoblast-like cells—Saos-2) as cell viability, cell proliferation, cell differentiation.
3.  Detecting of cell factors, promoting wound healing response.
4.  BG antibacterial activity.
5.  Detecting change in levels of inflammatory modulating molecules.
6.  Regarding the in vivo tests these involved: (a) dissolution of BGs in implanted liquids/tissues, (b) resorption of material particles based on size, (c) replacement of particles with new bone, (d) new tissue regenerated in the defects considered.

### 2.2. Inclusion/Exclusion Criteria

Inclusion criteria were:

- Clinical studies carried out on patients diagnosed with moderate to severe (chronic or aggressive) periodontitis, and at least presenting one periodontal intrabony defect grafted with BG (in particulate form, scaffold, composite, etc.) and treated by periodontal regenerative surgery.
- In vitro and in vivo studies considering the secondary outcomes of interest.

Exclusion otherwise.

### 2.3. Search Methods

The search strategy with appropriate keywords was carried out on three electronic databases (PubMed, Scopus, and Web of Science) with the following terms: Periodont* and bioactive glass. Data restrictions were applied from 1 January 2006 to 31 March 2021.

All the collected studies were considered by two independent reviewers (R.S. and C.B). During the first phase the eligibility of studies was assessed by screening both the title and the abstract, using the pre-specified inclusion and exclusion criteria. The studies not fitting the inclusion criteria were excluded. If disagreement on inclusion/exclusion could not be resolved, a further reviewer was used (V.C.).

Afterwards, the bibliographies of the considered studies were examined to identify additional missing relevant articles with the same methodology described above.

## 3. Results

A total of 340 articles were found through the three electronic databases; after removing the duplicates, a total of 150 articles were identified. The full text of the resulting 150 papers was screened, then articles were subdivided into: clinical studies, in vitro and in vivo studies, and many papers were excluded as they did not meet the inclusion criteria. At the end, a total of 51 papers were included in the current manuscript. Selected papers with in vitro and in vivo tests are presented in Section 3.1 and Section 3.2, respectively, and the list of clinical studies in Section 3.3.

**Figure 3:**
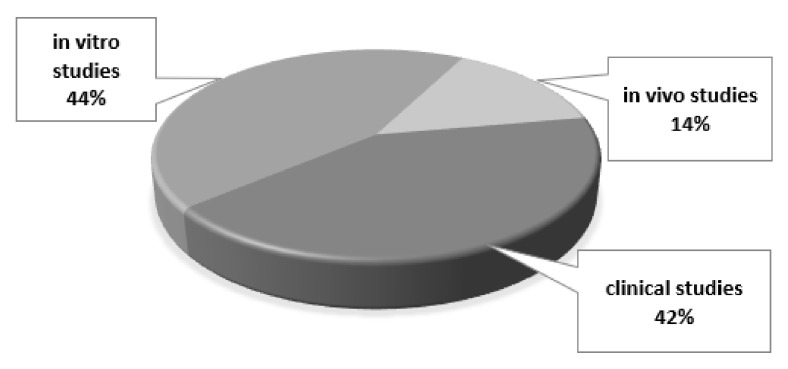
Articles distribution in clinical/in vitro/in vivo studies in periodontal regeneration with BGs, from 2006 to 2021.

**Figure 4:**
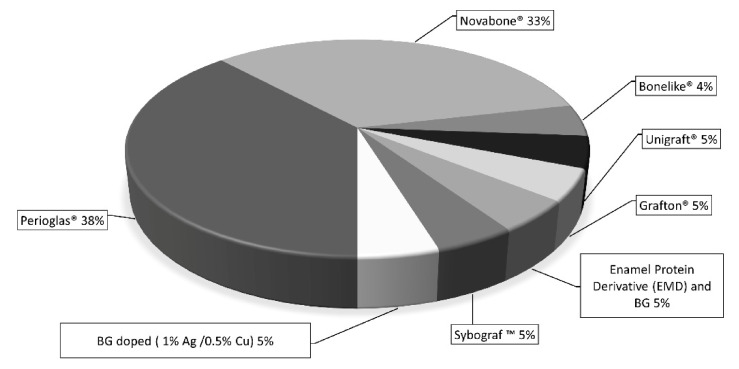
Different types of BGs used in clinical studies in periodontal patients from 2006 to 2021.

The analysis of the literature highlighted that only a few in vivo (in animal) studies were reported, whilst many in vitro studies were available (see Figure 3). With regard to clinical trials, the selected studies showed a considerable heterogeneity in terms of experimental design, aims, sample dimension and different BGs considered (Figure 4). Regarding the BG composition, most of the studies considered: 45S5 Bioglass, PerioGlas^®^ (a commercial product based on 45S5), and NovaBone dental putty^®^ (BG particles combined with a polyethylene glycol and glycerin binder).

**Figure 5:**
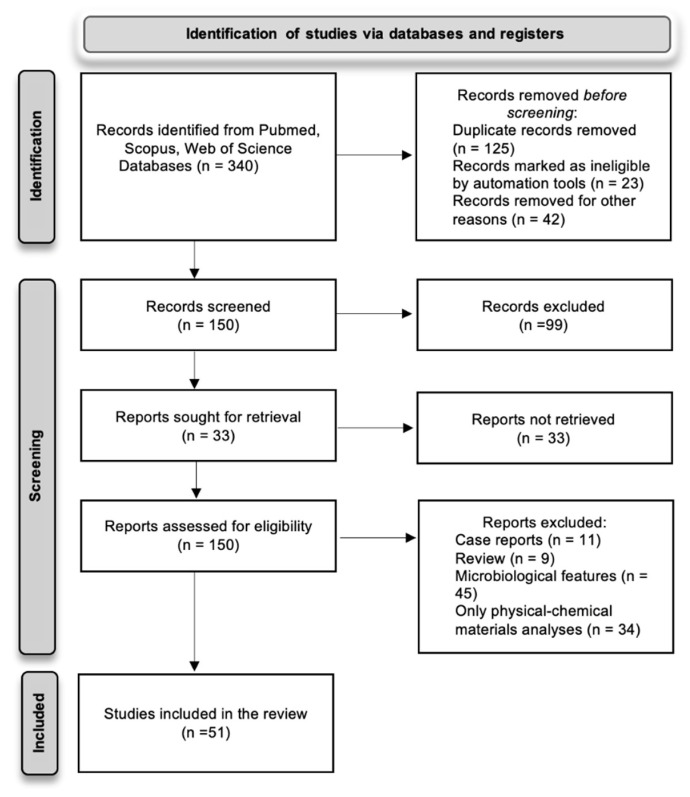
Flow diagram of the included articles.

The flow diagram of the included articles is illustrated in Figure 5.

### 3.1. In Vitro Studies

#### 3.1.1. BG Microparticles

Balamurugan et al. [52] evaluated the antibacterial activity and biological behavior of a bioactive glass obtained by a sol-gel process and containing silver. The dissolution kinetics of the silver-doped BG was preliminarily evaluated by means of SBF tests, demonstrating that the dissolution of silver ions from the BG was slow compared to the dissolution of other BG constituents, and that the material was able to release silicate species. Subsequently, the bacteriostatic and bactericidal capability of the glass was studied using a MG1655 strain of Escherichia coli, demonstrating that an inhibition of bacterial growth was obtained for specific concentrations of the BG. Moreover, this BG also elicited a rapid bactericidal action; such an action was exclusively attributed to the leaching of Ag^+^ ions from the BG. The slow silver ion release could allow a controlled and prolonged delivery of this antibacterial agent in clinical application. Considering the results obtained in in vitro test, this BG could be used even under anaerobic conditions such as in the periodontal pocket.

Expression of type 1 collagen (Col1α1, Col1α2), osteocalcin and alkaline phosphatase on human periodontal ligament fibroblasts (hPDLF) in contact with a BG conditioned medium (GCM) were analyzed by Varanasi et al. [53]. The GCM was made with (i) commercial 45S5 and (ii) an experimental BG. The bioactive glass particles were dissolved in the cell culture medium and, after 3 days, the resulting glass conditioned medium was measured in its ion concentrations using inductively coupled plasma mass spectrometry. GCM was used on human periodontal ligament fibroblasts cell cultures in contact for 16 days, to evaluate the amount of calcium deposited by staining with alizarin red, showing an increase in deep red color (increase in Ca), within the extracellular matrices. By means of RT-PCR, gene expression of type 1 collagen (Col1α1, Col1α2), osteocalcin, alkaline phosphatase, which favor the formation of mineralized tissue, was quantified. The favorable biological action on the extracellular matrices was also confirmed by the SEM analysis, which highlighted an abundant formation of bundles of collagen fibers in the cells in contact with GCM.

#### 3.1.2. BG Nanoparticles and Mesoporous BGs

Mesoporous bioactive glasses have been studied in vitro for the treatment of periodontal defects by several research groups (see Table 1).

Casarrubios et al. [54] studied mesoporous nanospheres with potential applications for periodontal treatment. The authors evaluated the cytocompatibility of such nanospheres containing ipriflavone (IP), a synthetic isoflavone that prevents osteoporosis. The mechanisms by which the nanospheres were incorporated within the MC3T3-E1 cells were identified using several inhibitors (cytochalasin B, cytochalasin D, chlorpromazine, genistein, and wortmannin). Besides, the particles resulted cytocompatible, as demonstrated also by studies of cell viability measured by adding propidium iodide in the samples. The analysis of the cell cycle and apoptosis by flow cytometry showed that the nanospheres did not induce changes in the cell cycle. The research evidenced the active incorporation of nanospheres by MC3T3-E1 osteoprogenitor cells that stimulated their differentiation into mature osteoblast phenotype with increased alkaline phosphatase activity in comparison with control cultures. The study demonstrated the biocompatibility and osteogenic behavior of IP-loaded bioactive nanoparticles to be used for periodontal augmentation purposes.

Bai et al. [55] investigated boron-containing mesoporous bioactive glass nanospheres (with average size of 60 nm); such nanospheres contained boron in increasing percentages (from 5 to 20 mol.%). Immersion tests in simulated body fluid (SBF) were performed, demonstrating an increase in the deposition of hydroxyapatite (HA) with a percentage increase in the amount of boron. Cell proliferation tests were performed with human periodontal ligament cells (hPDLC) and the results demonstrated good cell proliferation and no significant toxic effect.

Wu et al. [37] studied strontium containing mesoporous bioactive glass scaffolds. The analysis of ion release and mineralization of the scaffolds was performed according to the standardized Kokubo protocol. Additionally, proliferation, alkaline phosphatase (ALP) activity and osteogenesis/cementogenesis-related gene expression of periodontal ligament cells (PDLCs) on different kinds of scaffolds were investigated. The authors demonstrated that the incorporation of Sr into the glass scaffolds significantly stimulated ALP activity and osteogenesis/cementogenesis-related gene expression of PDLCs.

Jia et al. [56] studied porous bioactive mesoporous glass scaffolds, containing strontium (with Sr concentration of 5 mol.%). The authors investigated the mechanism of periodontal regeneration stimulated by strontium. The cells used were periodontal ligament stem cells (PDLC), in contact with the scaffolds to study the osteoblastic differentiation mechanisms stimulated by Sr in vitro. Several in vitro tests were performed. It was found that the epigenetic mechanism of splicing factor hnRNPL (a heterogeneous nuclear ribonucleoprotein L) participated in the osteogenesis processing of PDLCs stimulated by SrCl_2_.

Bioactive glass nanoparticles (BGNP), obtained by sol–gel using a modified Stober method, were studied by the research group of Carvalho et al. [57]. The authors isolated and characterized primary rat cementoblasts and evaluated their cellular response in a medium containing ionic compounds from dissolution of BGNP. The study showed that the ionic products from bioactive glass nanoparticles increased the viability of the cementoblasts, their mitochondrial activity, and cell proliferation. Indeed, BGNP induced cementoblasts to proliferate, thus pointing out that they could be used in cement regeneration.

#### 3.1.3. Scaffolds and Composites

Sowmya et al. [58] developed a nanocomposite scaffold based on chitin hydrogel and bioactive glass ceramic particles (nBGC). Human osteosarcoma cells (MG63) and human primary osteoblastic cells (POBs) were used for cellular studies. A cell attachment test and a cell proliferation test were performed by seeding MG63 and POB cells on the composite chitin/nBGC scaffolds. Biological tests showed that bioactive glass ceramic nanoparticles with chitin hydrogel were not toxic, as they did not release toxic substances for MG63 and POB cells. Moreover, from cell proliferation tests, the authors demonstrated that the scaffolds had good biocompatibility and could be used to regenerate periodontal bone in defects.

The same research group [59] performed further studies on scaffolds for periodontal bone and tissue regeneration. The authors developed a three-layer nanocomposite scaffold consisting of: (i) chitin—PLGA/nanobioactive glass ceramic (nBGC)/cementum protein 1 (cementum layer), (ii) chitin—PLGA/fibroblasts growth factor 2 as the periodontal ligament layer (PDL) and (iii) chitin—PLGA/nBGC/platelet-rich plasma-derived growth factors as the alveolar bone layer. The study involved an analysis of the cementogenic, fibrogenic, and osteogenic expression of human dental follicle stem cells (hDFCs) seeded on the tri-layer scaffolds, by flow cytometry. Cementogenic expression was studied by analyzing type 1 collagen (COL1), cementoblastoma-derived protein 1 (CEMP1), bone sialoprotein (BSP) in hDF in contact with the scaffold on day 7, 14, and 21. Fibrogenic differentiation was also analyzed by flow cytometry by analyzing the fibroblast surface protein (FSP), COL1, and periodontal ligament-associated protein 1 (PLAP1). The expression of anti-human runt-related transcription factor 2 (RUNX2), COL1, and anti-osteocalcin-human (OCN) were considered to study osteogenic expression. Alkaline phosphatase (ALP) analysis was also performed in hDFCs grown on the scaffolds. Cellular and molecular tests showed that the three-layer nanocomposites induce cementogenic, fibrogenic, and osteogenic differentiation of hDFCs comparable to cell differentiation.

Nanocomposites constituted by niobium- and zinc-doped bioglass-ceramic particles and chitosan were prepared and characterized by Uskoković et al. [60]. The authors characterized the nanocomposites from a physicochemical point of view (transmission electron microscopy, energy dispersion X-ray analysis, X-ray diffraction, and Fourier transform infrared spectroscopy). From a biological point of view, murine dental papilla cells (MDPc) were tested (similar to odontoblasts) in contact for 24 h, with bioglass-ceramic nanoparticles doped with Zn or Nb and undoped bioglass-ceramic nanoparticles, by cell viability assay. An antimicrobial activity test was also performed by diffusion of the agar (Trypsin Soy Agar) of colonies of Streptococcus mutans (UA159) in contact with doped bioglass-ceramic nanoparticles with niobium and zinc. While doping with Zn or Nb showed an interesting effect on cell viability, the Zn-doped glass-ceramic was less viable to odontoblasts than the control and the Nb-doped one.

A composite scaffold made of alginate and nanobioactive glass ceramic particles (nBGC, CaO-SiO_2_-P_2_O_5_ ternary system) was fabricated and characterized by Srinivasan et al. [61] based also on studies previously performed on nBGC [59]. A protein adsorption assay was performed, placing the scaffolds in contact with the minimum essential media (MEM), for a duration from 30 min to 6 h, and quantifying the total proteins adsorbed with the bicinchoninic acid (BCA) assay (which is based on the reduction of Cu^2+^ to Cu^1+^, the extent of the reduction is proportional to the protein adsorbed on the scaffold). The in vitro biomineralization of the scaffolds was also evaluated by embedding the samples in simulated body fluid (SBF), according to Kokubo [62], for 7, 14, 21, and 28 days. The possible formation of apatite on the scaffold was evaluated by means of SEM and Energy Dispersive Spectroscopy (EDS); the bioactivity of the samples was further confirmed by X-ray diffractometry (XRD). The authors verified that the scaffolds were biocompatible with human periodontal ligament fibroblast (hPDLF) and osteosarcoma cells (MG-63). The hPDLF cells also behaved as osteoblasts showing enhanced alkaline phosphatase activity. The cytocompatibility, good cell adhesiveness, and proliferation suggested the possible use of such scaffolds for the regeneration of periodontal tissue.

In an experimental in vitro study, Esfahanizadeh et al. [63] investigated the anti-biofilm activity of a BG doped with zinc compared to 45S5, on periodontal pathogens. The zinc-doped BG was produced by means of a sol-gel technique. The antibiofilm potential of the two materials was measured. Both BGs reduced the biofilm formation, but the antibiofilm activity of the Zn-doped BG was significantly stronger than that of 45S5.

Barrier membranes are used in periodontal applications with the aim of supporting periodontal regeneration by physically blocking migration of epithelial cells. Such devices are often referred as guided tissue regeneration (GTR) membranes. Caridade et al. [64] developed biocompatible and biodegradable composite membranes by combining particles of poly (d,l-lactic acid) PDLL and Bioglass^®^, studying both the mechanical properties and the biological properties of the membranes. Cytocompatibility by MTS 3-(4,5-dimethylthiazol-2-yl)-5-(3-carboxymethoxyphenyl)-2-(4-sulfophenyl)-2*H*-tetrazolium assay was performed after 1, 3, and 7 days and cell proliferation using the PicoGreen kit dsDNA for DNA quantification was evaluated on osteoblast-like cells (Saos-2). The authors observed that both the metabolic activity and the cell viability increase with increasing culture time in contact with the material. In fact, after 7 days of contact with the BG/membrane system, it is possible to observe the best data in terms of cytocompatibility. Statistical data, on the other hand, show no significant changes in cell proliferation with BG-integrated membranes.

The biological behavior of bilayer membranes (of cellulose acetate) containing bioactive glass nanoparticles modified with boron was studied by Moonesi et al. [65]. BG nanoparticles were studied by SEM and Fourier transform Raman (FT-Raman) analysis to obtain information on the structure and characteristics of the particles. The bilayer membranes were morphologically characterized by scanning electron microscopy (SEM) and the contact angle was measured by means of a goniometer. In vitro membrane degradation and dissolution tests were performed to evaluate the ionic dissolution of BG for Si^4+^, Ca^2+^, and B^+3^ ions. In order to investigate the membrane cytocompatibility, cell adhesion, diffusion, and proliferation, tests (Alamar blue cell viability test) were performed on human dental pulp stem cells (hDPSc) in contact with the membrane. Furthermore, ALP activity and intracellular calcium quantities were quantified after 7 and 14 days of incubation. The authors found an increase in both the indicators, showing how hDPSc on bilayer membranes containing boron-modified bioactive glass nanoparticles proceeded to osteogenic differentiation. Moreover, the authors demonstrated that bioactive glass modified with 7% boron was a candidate material for use in guided bone regeneration (GBR).

Mota et al. [66] produced and characterized a novel membrane for guided tissue and bone regeneration containing chitosan with bioactive glass nanoparticles (BG-NP). In addition to physical characterization, tests to evaluate tensile strength and tensile modulus, and the SBF test to evaluate bioactivity were carried out. The membranes were biologically tested in contact with human periodontal ligament cells (hPDL) and human bone marrow stromal cells (hBMSC). Cell viability (Alamar Blue test), cell proliferation, and evaluation of calcium content after cell seeding on the membrane were performed to obtain information on the formation of the mineralized matrix. Composite membranes were shown to have bioactive potential with the development of an apatite layer after 5 days in SBF. According to the authors, the produced samples favored metabolic activity and cell proliferation in addition to cellular mineralization, and the composite membranes could be used in guided tissue regeneration in periodontal applications, given their ability to induce osteo-regeneration.

Ruiz-Clavijo et al. [67] investigated binary glasses (CaO-SiO_2_) for potential application in guided tissue regeneration membranes for periodontal repair. The authors studied several calcium-based reagents for producing the two components-glass by sol-gel. Bioactivity was evaluated by means of possible formation of a surface layer of hydroxyapatite, by ionic dissolution. Biocompatibility was assessed using a MTT (indirect cytotoxicity) test on composite membranes containing BG particles and chitosan in contact with human MG63 osteosarcoma cells. The authors demonstrated that composite membranes with glasses derived from calcium ethoxide and chitosan could be candidates for periodontal regeneration.

Three-layered functionally graded membranes for periodontal regeneration were prepared by Shah et al. [68]. The membranes contained various concentrations of bioactive glass nanoparticles (BG-NP) in each layer depending on the function of each layer (bone or tissue regeneration). The lower layer contained 50% wt. BG-NP, thus mimicking bone, the middle layer had 25% wt. BG-NP and in the upper non-porous layer BG-NPs were not added. Cytocompatibility and cell adhesion analysis was performed on pre-osteoblastic cells of MC3T3-E1 mice. Cell proliferation (Alamar blue test) revealed the biocompatible nature of the membranes, while cell adhesion occurred mainly on the lower layer of the membrane surface.

Sunandhakumari et al. [69] designed membranes for guided tissue regeneration, made of PCL and BG particles. Physico-chemical analysis (scanning electron microscopy, Fourier transform infrared spectroscopy, X-ray diffraction) was performed to characterize the membranes. Biological analysis (MTT test), performed on a mouse fibroblast cell line [L-929] in contact with the extracts of the samples, showed the non-cytotoxicity of the membranes, pointing out how the membrane manufactured with 2% BG had produced higher cell attachment and higher percentage of cell viability.

Beketova et al. [70] studied a new bioactive glass/ceramic dental composite, in particular the authors investigated the effect of a laser treatment to accelerate the formation of the surface layer of hydroxyapatite (HA). The HA film was physico-chemically characterized by X-ray diffraction analysis (XRD), Fourier transformed infrared analysis (FTIR), micro-Raman analysis, scanning electron microscopy and energy dispersion X-ray microanalysis (SEM-EDS). The study checked also for any changes in the chemical composition of the composite, before and after laser irradiation. Finally, cytocompatibility was assessed by cell viability test (MTT) after 24, 48, or 72 h, with periodontal ligament fibroblasts (hPDLF), human gingival fibroblasts (hGF), and Saos-2 osteoblasts. However, the laser did not increase gingival and periodontal proliferation but only osteoblastic proliferation.

A composite formed by bioactive glass and polycaprolactone (PCL), in the form of a scaffold that mimicked bone architecture, was created by Granel et al. [71]. The authors evaluated both the biological potential in vitro using primary rat osteoblasts (RPO) and in vivo in a mouse model of cranial defects. Cell viability and cell proliferation were determined by the XTT test (2,3-Bis-(2-methoxy 4-nitro-5-sulfophenyl)-2*H*-tetrazolium5-carboxanilide salt). The osteogenic potential of the composite was evaluated by the alkaline phosphatase activity assay, and by the Runx2 immunoassay, FAK, phospho-FAK (Y397), GAPDH; additionally, other markers of the osteoblastic lineage differentiation were investigated. The authors found that such BG-PCL hybrid scaffolds promoted cell adhesion and diffusion and that the dissolution products from the scaffolds preserved the viability and osteogenic potential of the cells.

Meneses et al. [72] investigated the cytotoxicity and cell modulation effects of gutta-percha/niobium phosphate glass on human periodontal ligament fibroblasts (hPDLF). The assay of cell viability (AlamarBlue) was performed and gene expression of type I collagen and cement protein by quantitative reverse transcription polymerase chain reaction was determined. The authors demonstrated that the material was cytotoxic to hPDLFs in both pure extracts and dilutions 1/5 and 1/10. Moreover, the gene expression of type I collagen was down-regulated, while the expression of the cement 1 protein remained unaltered. Concluding that, the addition of glass niobium phosphate to the gutta-percha had a negative action on the cell proliferation of hPDLF.

Theocharidou et al. [73] tested in vitro two types of ceramic composite scaffolds made by leaching water non-soluble particulates, on the surface of modified ceramic disks (MCD), with a mixture of bioactive glass–ceramic. The authors studied the surface characteristics of the scaffolds after 10 days of incubation with or without human periodontal ligament fibroblasts (hPDLF), and found that cell attachment and function could decrease the surface’s porosity, thus consequently affecting hPDLF proliferation. 

#### 3.1.4. Bulk BG

The analysis of cell viability and proliferation on different types of cells (cementoblasts, osteoblasts, and fibroblasts) after contact with the dissolution products from a bioactive glass produced by sol-gel was the goal of the study by Carvalho et al. [74]. In the MTT and Trypan Blue test, an increase in mitochondrial activity was observed in all cell types studied, and to a greater extent in the cementoblasts. This study suggests a positive effect of bioactive silica particles on cementoblasts, making this material useful in fabricating membranes for cement tissue engineering.

Wen et al. [75] developed a bioactive glass based on the xSiO_2_-CaO-P_2_O_5_ system (x = 30, 45, 60 and 90 mol.%, Ca/P = 1.67) synthesized with a sol–gel method. Physico-chemical characteristics (ability to form apatite) and biological behavior were evaluated. The cytotoxicity test, Cell Counting Kit-8 (CCK8), was performed on human periodontal ligament (hPDLC) cells incubated with different concentrations of glass extract at 1, 3, and 7 days. The results demonstrated no inhibitory effects on cell growth and excellent ability to form apatite. The BGs also showed to have a connected system of internal mesoporous structures.

**Table 1:** Summary of in vitro studies.

References	Materials	Type of Cells and Tests
Balamurugan et al. [52]	Sol-gel BG containing silver	*E. coli* (MG1655) Simulated body fluid (SBF) test Antimicrobial activity
Varanasi et al. [53]	(i) commercial 45S5 and (ii) an experimental BG	Human periodontal ligament fibroblasts (hPDLF) Cell proliferation assay Osteocalcin and alkaline phosphatase gene expression (quantitative PCR) Protein expression assays (BCA assay) Mineralization Assay (Alizarin Red)
Casarrubios et al. [54]	mesoporous BG nanospheres	MC3T3-E1 pre-osteoblasts NanoMBG incorporation (flow cytometry) Cell viability, Cell-Cycle Analysis, apoptosis detection (flow cytometry) Intracellular Reactive Oxygen Species (ROS) and Intracellular Calcium Content (flow cytometer) Alkaline phosphatase (ALP) activity Mineralization Assay (Alizarin Red) Oxidative stress (Interleukin 6 detection)
Bai et al. [55]	Boron-containing mesoporous bioactive glass nanospheres (with average size of 60 nm) Boron from 5 to 20 mol.%	Human periodontal ligament cells (hPDLCs) Cell viability [CCK-8] Simulated body fluid (SBF) test
Wu et al. [37]	Strontium containing mesoporous BG scaffolds	Human periodontal ligament cells (hPDLCs) Simulated body fluid (SBF) test Alkaline phosphatase (ALP) activity Protein expression assays (BCA assay) Collagen type I (COL1), osteopontin (OPN), runt-related transcription factor 2 (RUNX2) and cementum protein 1 (CEMP1) gene expression
Jia et al. [56]	Porous mesoporous BG scaffolds, containing strontium (Sr 5 mol.)	Periodontal ligament stem cells (PDLCs) Epigenetic mechanism evaluation
Carvalho et al. [57]	Bioactive glass nanoparticles (BGNP)	Osteoblasts rat calvaria Gingival fibroblasts wistar rats Cementoblast wistar rats Cell viability (Trypan blue assay)Mitochondrial activity [MTT assay] Cell proliferation (BrdU assay) Alkaline phosphatase (ALP) activity Mineralization nodules (Von Kossa staining) Protein expression (Western blot analysis and reverse transcription polymerase chain reaction (RT-PCR)
Sowmya et al. [58]	Nanocomposite scaffold based on chitin hydrogel and bioactive glass ceramic particles (nBGC)	Human osteosarcom a cell line (MG63) Human primary osteoblasts cells (POB) Simulated body fluid (SBF) test Cell viability (Alamar blue assay) Cell adhesion (by SEM) Cell proliferation (DAPI staining) POB maturation and mineralization
Sowmya et al. [59]	Three-layer nanocomposite scaffold consisting of: (i) chitin—PLGA/nanobioactive glass ceramic (nBGC)/cementum protein 1, (ii) chitin—PLGA/fibroblasts growth factor 2 and (iii) chitin—PLGA/nBGC/platelet-rich plasma-derived growth factors	Human dental follicle stem cells (hDFCs) Cementogenic, fibrogenic, osteogenic, differentiation (by flow cytometry) Alkaline phosphatase (ALP) activity Biomineralization (SEM)
Uskoković et al. [60]	Nanocomposites: niobium- and zinc-doped bioglass-ceramic particles and chitosan	Odontoblast-like MDPC-23 cells Cell viability (CellTiter-Blue assay)
Srinivasan et al. [61]	Composite scaffold: alginate and nanobioactive glass ceramic particles (nBGC, CaO–SiO_2_–P_2_O_5_ ternary system)	Human periodontal ligament fibroblast (hPDLF) Human osteosarcoma cell line (MG-63) Protein adsorption studies (bicinchoninic acid assay-BCA) Biomineralization (simulated body fluid—SBF) Cell viability assay (Alamar blue) Alkaline phosphatase (ALP) activity Cell proliferation (DAPI staining)
Esfahanizadeh et al. [63]	BG doped with zinc compared to 45S5	Antibiofilm activity
Caridade et al. [64]	Composite membranes: poly (d,l-lactic acid) PDLL and Bioglass^®^	Saos-2 cells Cell viability (MTS assay) Cell proliferation (PicoGreen test) SEM morphological evaluation
Moonesi et al. [65]	Bilayer membranes (of cellulose acetate) containing BG nanoparticles modified with boron	Human dental pulp stem cells (hDPSCs) Dissolution (by inductively coupled plasma mass spectrometry—ICP-MS) Simulated body fluid (SBF) test SEM morphological evaluation Cell viability (Alamar blue assay) Mineralization Assay (Alizarin Red) Alkaline phosphatase (ALP) activity Cell migration (Confocal laser scanning microscopy-CLSM)
Mota et al. [66]	Membrane: chitosan with bioactive glass nanoparticles (BG-NP)	Human periodontal ligament cells (hPDL) and human bone marrow stromal cells (hBMSC). Cell viability (Alamar Blue test) Cell proliferation Evaluation of calcium content
Ruiz-Clavijo et al. [67]	Binary glasses (CaO-SiO_2_)	Human osteosarcoma cell line (MG63) Cell viability assay (MTT test)
Shah et al. [68]	Three-layered functionally graded membranes, with various concentrations of BG nanoparticles	Murine pre-osteoblasts cell line (MC3T3-E1) Cell viability (Alamar blue assay) Cell adhesion (by SEM)
Sunandhakumari et al. [69]	Membranes: polycaprolactone (PCL) and BG particles	Murine fibroblast cell line (L-929 Cell viability (XTT assay))
Beketova et al. [70]	BG/ceramic dental composite	Periodontal ligament fibroblasts (PDLFs) Human gingival fibroblasts (HGFs) Saos-2 osteoblasts Simulated body fluid (SBF) test Cell viability test (MTT)
Granel et al. [71]	BG and PCL	Rat primary osteoblastic (RPO) cells Cell viability assay (XTT test) Cell proliferation (CyQUANT NF assay) SEM morphological evaluation Cell signaling (immunoassay for Runx2, FAK, phospho-FAK (Y397), GAPDH) Alkaline phosphatase activity assay
Meneses et al. [72]	Gutta-percha/niobium phosphate glass	Human periodontal ligament fibroblasts (hPDLF) Cell viability (AlamarBlue) Gene expression of type I collagen and cement protein by quantitative reverse transcription polymerase chain reaction
Theocharidou et al. [73]	BG/ceramic composite scaffolds	Human periodontal ligament fibroblasts (hPDLF) Cell attachment (by SEM)
Carvalho et al. [74]	BG by sol-gel	Cementoblasts (from the molars extracted from Wistar male rats) Osteoblasts (from calvaria of neonatal Wistar rats) Neonatal fibroblasts (from hearts of Wistar rats) Cellular viability (Trypan Blue assay, MTT assay)
Wen et al. [75]	BG based on the xSiO_2_-CaO-P_2_O_5_ system	Human periodontal ligament cells (hPDLCs) Simulated body fluid [SBF] test Cell viability (CCK-8)

### 3.2. In Vivo Studies

In vivo animal studies found in the literature are quite heterogeneous (see Table 2).

Carvalho et al. [76] evaluated periodontal regeneration in animal-model study (9 mongrel dogs), by means of a randomized-controlled study on periodontal intraosseous 3-wall defects surgically created on the mesial and distal aspect of the mandibular first molar. Periodontal defects were treated with: (i) BG particles (Perioglas^®^); (ii) plasma rich in platelets (PRP); (iii) BG and PRP; (iv) control, with a 90-days follow-up. Through a histomorphometric and histological analyses, a superior area of new bone was observed in PRP + BG and BG when compared to the other groups (control and PRP, respectively). No statistically significant differences were observed in the remaining histomorphometric parameters (length of sulcular and junctional epithelium, connective tissue adaptation). The authors concluded that the association of PRP with BG did not have any additional effect of periodontal regeneration on intraosseous defects in dogs.

Felipe et al. [77] investigated bone regeneration using a canine animal model. Two-wall intrabony periodontal defects were surgically induced on the mesial surfaces of the mandibular third premolars and first molars bilaterally in six dogs. The defect regenerative treatment was performed with a bioabsorbable membrane in association with bioactive glass of different sizes (namely Perioglas^®^ and BioGran). The study comprised 4 groups: (i) membrane and Perioglas^®^ (particles size 90 to 710 microns); (ii) membrane and BioGran (particles size 300 to 355 microns); (iii) membrane alone; (iv) negative control. The follow up was 90 days, a histomorphometric and histological evaluation of the regenerative surgery was performed. Histomorphometric measurements highlighted better regenerative results in group 1. However, the use of BG particles in the animal model seemed to promote bone formation; there were greater areas of mineralized bone in groups (i) and (ii) compared to groups (iii) and (iv).

An investigation of bone formation was conducted in dogs by Lee et al. [78]. One-wall critical intraosseous periodontal (1-wall defect) defect was induced on the mesial or distal aspect of the mandibular second and fourth premolars of 5 beagle dogs. The regenerative treatment was performed using four different strategies: (i) an amorphous calcium phosphate glass cement with collagen membrane (CM), (ii) biphasic calcium phosphate with CM, (iii) CM alone, and (iv) surgical flap operation only (control group, not grafted). A histological evaluation of the block sections of the defect sites was performed. An automated image analysis system was used for histomorphometric analysis. The periodontal regeneration was evaluated 2 months after surgery, showing that the calcium phosphate glass cement and the biphasic calcium phosphate stimulated more bone formation than the collagen membrane or the control group. The calcium phosphate glass cement slightly contributed to regeneration of the periodontal apparatus.

After performing the physico-chemical analysis and cytocompatibility test of a three-layer nanocomposite scaffold (previously presented in the “In vitro studies” section), Sowmya et al. [59] also evaluated the in vivo biocompatibility. The scaffolds (with and without grow factors) were implanted in surgically created maxillary 6 × 5 mm wide and 4 mm deep periodontal defects in 12 rabbits. The animals were randomly subdivided into 4 groups: group I (sham/negative control), group II as guided tissue regeneration (positive control), group III used three-layer nanocomposite scaffolds, and group IV used three-layer nanocomposite scaffolds with growth factors. The histomorphometric assessments of tissue regeneration were performed at 1 and 3 months after surgery. After 3 months the authors highlighted the complete defect healing in the case of scaffold with growth factors (group IV); formation of new cementum, fibrous periodontal ligament, and alveolar bone were observed. The root surface remained exposed in group I, new tissue formation with incomplete healing was observed in group II (guided tissue regeneration membrane—positive control), and in group III (scaffold without growth factors) new thin and irregular cementum with fibrous PDL formation was observed.

Zhang et al. [79] evaluated periodontal regeneration in osteoporotic rats. Bilateral fenestrations at the buccal aspect of the mandibular first molar (2.8 mm in length, 1.4 mm in height and < 0.5 mm in deep) were induced. The study was performed using three regenerative strategies to obtain periodontal regeneration: (i) unfilled defects as control, (ii) a BG scaffold (BG: CaO-P_2_O_5_-SiO_2_) and (iii) a scaffold made of BG also containing strontium. After 28 days the animals were sacrificed, and the regeneration evaluated through micro-CT and histomorphometric analysis. Periodontal fenestration defects treated with Sr-BG scaffolds showed greater new bone formation (46.67%) when compared to BG scaffolds (39.33%) and control unfilled samples (17.50%). Thus, such scaffolds resulted to be the more promising, especially for patients suffering from a combination of both periodontal disease and osteoporosis.

The study by Granel et al. [71], already examined in the previous paragraph (“In vitro studies”), dealt with BG-PCL hybrids in the form of scaffolds, implanted in bone defects (3.5 mm in diameter) created at each side of the parietal bone using a tissue punch in rat calvaria of 14 mice (8 used for the treatment and 6 as control—shame hole). An analysis was performed at 0-, 30-, 60-, and 90-days post-surgery, by X-ray micro-computed tomography (micro-CT), and after 90 days the animals were sacrificed. The bone regeneration was investigated by micro-CT and histomorphology after a 90-day follow-up. The study demonstrated a higher bone ingrowth with BG-PCL scaffolds, the vascularization of defects, and the complete chemical conversion of the remaining BG-PCL into a bone-like mineral, while in control defects the bone regeneration share was less than one half.

Shah et al. [68], besides the in vitro study, examined the same material (functionally graded membranes for periodontal regeneration) in vivo, in eight healthy adult Wistar rats. The authors evaluated the biocompatibility of the membranes for periodontal regeneration up to 35 days. In this case no bone defect was generated; instead, subcutaneous pockets were created and the membrane with bioactive glass was placed into the same pockets. After 35 days the animals were sacrificed, and histological analysis (hematoxylin and eosin) was performed. The membranes had no systemic side effects and rapid formation of connective tissue at membrane-tissue interface was observed, thus pointing out their biocompatibility.

**Table 2:** Summary of in vivo studies (in animal).

References	Materials	Animal and Follow-Up
Carvalho et al. [76]	(i) BG (Perioglas^®^, 90–710 µm); (ii) plasma rich in platelets (PRP); (iii) BG and PRP; (iv) control	9 mongrel dogs Follow-up: 90 days
Felipe et al. [77]	(i) membrane and Perioglas^®^ (particles size 90 to 710 microns); (ii) membrane and BioGran (particles size 300 to 355 microns); (iii) membrane alone; (iv) negative control	6 dogs Follow-up: 90 days
Lee et al. [78]	(i) an amorphous calcium phosphate glass cement with collagen membrane (CM), (ii) biphasic calcium phosphate with CM, (iii) CM alone and (iv) surgical flap operation only (control group, not grafted)	5 beagle dogs Follow-up: 60 days
Sowmya et al. [59]	Nanocomposite scaffold (chitin hydrogel and bioactive glass ceramic particles—nBGC)	12 rabbits Follow up: 30 and 90 days
Zhang et al. [79]	(i) unfilled defects as control, (i) a BG scaffold (BG: CaO-P_2_O_5_-SiO_2_) and (iii) a scaffold made of BG also containing strontium	15 osteoporotic rats Follow-up: 28 days
Granel et al. [71]	Bioactive glass and polycaprolactone (PCL)	14 rats (calvaria) Follow-up: 30, 60, 90 days
Shah et al. [68]	Three-layered functionally graded membranes: lower layer with 50% wt. bioactive glass nanoparticles (BG-NP), middle layer 25% wt. BG-NP and upper layer no BG-NPs	8 wistar rats Follow-up: 35 days

### 3.3. Clinical Studies

Humagain et al. [80] (Table 3) carried out a randomized controlled study aimed at analyzing the effect of BG particles in the treatment of class II mandibular furcation defects. In 16 healthy patients suffering from class II mandibular furcation defects, 10 defects were randomly treated with open flap debridement (OFD) and 10 defects with OFD and a BG particulate (PerioGlas^®^-test sites). On 4 of the 16 enrolled patients, the study had a split-mouth design. The study showed better results on vertical and horizontal defect regeneration using BG together with flap surgery than treatment with the only OFD with a follow-up of 6 months.

Keles et al. [81] carried out a split-mouth randomized controlled study on deep periodontal intraosseous defects (PPD ≥ 6 mm) treated with platelet pellet (PP)—PP has a higher platelet content than platelet-rich plasma (PRP)—or a BG (PerioGlas^®^—US Biomaterials Corp., Alachua, FL, USA), and guided tissue regeneration (GTR) principles (absorbable GTR polylactic acid membrane). The study included 15 healthy patients with chronic periodontitis with a 6-month follow up. The clinical and radiological comparison between the differently treated defects did not show statistically significant differences with regard to periodontal attachment and bone regeneration 6 months after surgery. The same clinical results were obtained by several other studies.

Demir et al. [82] performed a clinical study on 29 systemically heathy patients suffering from chronic periodontitis and almost one interproximal defect with PPD ≥ 6 mm and radiographic evidence of vertical alveolar bone loss. The randomized controlled trial was aimed at evaluating the healing of intra osseous defects using a BG graft (Unigraft^®^, 200–420 μm) with and without (control) PRP supplement during a 9-month follow up. The two treatments did not show statistical differences suggesting that the BG alone was effective in the treatment of the intrabony defects. Cetinkaya et al. [83] considered eleven healthy patients, suffering from chronic periodontitis. Patients were randomly assigned to be treated with a combination of PP covered with a bioabsorbable polylactic acid barrier membrane (BM) or BG (PerioGlas^®^) covered with the same BM in contra-lateral dentition areas, using a split mouth design. The long-term efficacy of platelet pellet mixed with a barrier membrane is similar to the combination of bioactive glass graft material and barrier membrane, suggesting that results obtained with both treatment approaches could be maintained over a period of 5 years. Moreover, PRP in combination with a BG (PerioGlas^®^) or the BG alone were used by Kaur et al. [84] in a study on periodontal regeneration. The split-mouth study considered ten healthy patients suffering from chronic periodontitis with interproximal periodontal intraosseous defects surgically randomly treated with PRP + BG or BG alone, with a 6-months follow up. Both therapies resulted in significant PPD reduction, clinical attachment level (CAL) gain and defect fill. The association of PRP + BG provided good soft tissue clinical response, but no significant differences for the intraosseous defect regeneration.

Ten healthy patients with chronic periodontitis and vertical periodontal intraosseous defects were split-mouth treated with regenerative periodontal surgery and demineralized freeze-dried bone (DFDBA) in putty form (Grafton^®^) or with bioactive glass (PerioGlas^®^) [85]. The study showed clinical and radiological favorable results with both approaches 12 months after surgery. However, DFDBA showed better significant reduction in PPD, CAL gain, and a greater percentage of bone fill when compared to those of bioactive glass.

Twenty-five healthy patients, suffering from chronic periodontitis with deep intraosseous defects, were randomly treated with regenerative periodontal surgery using enamel matrix protein derivative (EMD) alone (control) or with a combination of EMD and bioactive glass [86]. The study had a 4-year follow-up and PPD, CAL, and gingival recession (GR) were considered as clinical outcomes. After evaluations carried out at baseline, 1, and 4 years the authors concluded that there were a statistically significant PPD reduction and CAL gain that could be maintained over a period of 4 years with both the treatments; no statistically significant differences at 1 and 4 years were found between control and test groups. Therefore, both regenerative modalities could be performed as post-surgical treatment for up to 4 years.

Kumar et al. [87] considered 10 heathy patients affected from aggressive periodontitis with a split-mouth designed study on 20 periodontal intrabony defects. A composite BG (Bonelike^®^, a glass reinforced HA with α and β forms of tricalcium-phosphate) was placed randomly in the test site using the OFD surgery; as an alternative, only the OFD surgical approach was carried out in control sites. PPD, CAL, GR were considered as soft tissue parameters, and 3D CT was used to obtain hard tissue parameters (bony defect depth, linear bone growth, alveolar crestal level, defect volume, and percentage defect fill) at baseline and 6 months after surgery. The test group (i.e., composite BG + OFD) showed significant better clinical and radiographic outcomes than OFD alone.

The aggressive periodontitis was also considered by Satyanarayana et al. [88] in a randomized controlled and split-mouth designed trial on twelve healthy patients. The study considered a three-walled intraosseous defect treated with periodontal surgery and a BG (PerioGlas^®^) graft, or only with surgery (control group). The results showed significant clinical improvements in bone defects treated with BG 12 months after surgery: in particular, in PPD, CAL and bone defect depth, measured by periapical radiograph, as the distance from alveolar crest to defect base.

Subbaiah and Thomas [89] also designed a study to compare the periodontal surgery alone with periodontal surgery in combination with a graft. A BG (PerioGlas^®^) was used as a replacement bone graft in the clinical study. Eight healthy patients, with collateral intrabony defects, were enrolled in a split-mouth designed study. Periodontal defects were randomly grafted with BG (PerioGlas^®^) or treated by the only OFD surgery (control group). Milestones were performed at 3, 6, 9 months to evaluate the effectiveness of adding the BG graft. The study showed that OFD surgery in combination with BG grafting was clinically favorable, allowing a significant improvement in the defect bone filling in comparison to the same obtained with the OFD alone.

Mistry et al. [90] compared the regeneration obtained in intrabony defects using randomly OFD alone (control group) or OFD + BG or OFD + hydroxyapatite (HA), or OFD + BG (50%) + HA (50%) in 22 healthy patients with 28 intrabony periodontal defects. The 28 intrabony defects were equally distributed (i.e., seven defects) into four groups. None of the patients received two or more grafts. The randomized controlled study was aimed at evaluating the soft and hard tissue responses through a clinical and radiographic comparison. Favorable clinical results in all the treatment groups, as CAL gain and PPD reduction, were obtained after 6 months. However, OFD + BG and OFD + BG + HA synthetic bone graft implanted sites showed significant bone fill, more than HA alone and un-implanted control sites.

In the same field, Lysienko and Borysenko [91] carried out a controlled, randomized study in 47 healthy patients suffering from periodontitis. The periodontal pockets were treated by periodontal surgery in combination with the BG graft. The BG was doped with silver and copper to add antibacterial properties. The BG was hydrated in glycosaminoglycans-based solution and was used with a bioresorbable membrane in test sites with surgery; in the control sites a DFDBA graft and the same membrane were used. The study showed a better, not significant, clinical outcome for PPD, and CAL (6 and 12 months after surgery) in the test group. A significant higher densitometry in interdental septs in the test group was highlighted 12 months after surgery. However, both the treatments showed significant regenerative clinical outcomes.

Slezák et al. [92] also designed a study using a combination of materials similar to those used by Lysienko and Borysenko [91]. It was a preliminary non controlled study aimed at evaluating the use of BG in addition to an absorbable binder, which was a combination of polyethylene glycol and glycerin (NovaBone Dental Putty^®^) for the treatment of intraosseous periodontal defects. The clinical study hired 10 patients suffering from chronic periodontitis. The enrolled patients were treated with periodontal surgery in combination with the glass-based bioactive material graft. Clinical parameters—such as PPD, GR, loss of attachment—were monitored for 10–56 weeks. The study highlighted a significant improvement in clinical parameters, in particular PPD reduction and periodontal attachment gain ware recorded.

Many authors have investigated this glass-based bioactive material (NovaBone^®^) in clinical studies. Grover et al. [93] also designed research using the same combination of materials used by Slezak et al. [92]. The authors performed a study using periodontal surgery and the Novabone Dental Putty^®^ in the treatment of intraosseous periodontal defects in twelve healthy subjects with chronic periodontitis. The regenerative results were considered 3 and 6 months after surgery. The study highlighted both a significant PPD reduction and a periodontal attachment level gain. Asmita et al. [94] performed a randomized, controlled trial to study the regeneration of horizontal class II furcation defects (mandibular molars) on 28 healthy patients suffering from chronic periodontitis. Forty furcation defects were randomly treated with periodontal surgery and NovaBone Dental Putty^®^ graft or surgery and BG (PerioGlas^®^) graft to fill the furcations. Clinical and cone beam computed tomography (CBCT) analyses were performed 6 months after the surgery. The study highlighted no significant difference between the two treatment groups, i.e., clinical and radiological regenerative outcomes were similar. The BG putty (NovaBone Dental putty^®^) in comparison with platelet rich fibrin (PRF) was also tried on mandibular horizontal class II furcation in a 9-month follow-up study. Biswas et al. [95] carried out a randomized, controlled trial on 15 healthy patients with 20 furcations subdivided into two surgical treatment groups. The defects were treated by periodontal surgery and grafted with NovaBone in the test group and PRF in the control group. The clinical comparison between the groups of treatment for PPD, CAL, and recession (REC) showed more effective regenerative outcomes at test sites. The NovaBone^®^ alone or mixed was compared with PRF to study periodontal intraosseous defects regeneration. Naqvi et al. [96] performed a randomized, controlled split-mouth trial on 20 healthy patients suffering from chronic periodontitis. The patients were treated by periodontal surgery and grafted with NovaBone^®^ in combination with PRF on one side or grafted with NovaBone^®^ alone (control) on the other side with a follow up of 9 months. Both the treatments resulted effective to regenerate the periodontal defects. However, CAL gain and defect fill at 6 and 9 months after surgery were significantly more effective using the BG putty in combination with PRF. The same clinical results were obtained by Saravanan et al. [97]. The periodontal surgery in combination with NovaBone Putty^®^ or nanocrystalline hydroxyapatite (Nc-HA) (Sybograf ™) grafts was performed to regenerate 20 intrabony periodontal defects in 20 patients suffering from chronic periodontitis. The study by Koduru et al. [98] was aimed at evaluating the periodontal regeneration considering periodontal indices and intraoral periapical radiographs. The study had milestones at 3, 6, and 9 months after surgery. Both the graft materials achieved favorable clinical outcomes. Nc-HA displayed slightly superior, non-significant, effects on clinical parameters with a 9-month follow up. As far as the comparison between the clinical outcomes obtained with the periodontal surgery in combination with HA or BGgraft was concerned, this study seemed to show opposite clinical outcomes from those obtained by Mistry et al. [90] and Lysienko and Borysenko [91].

The BG putty NovaBone^®^ was considered also in gingival recession (GR) surgical treatment as a sub-epithelial graft. Ten healthy patients with bilateral and comparable Miller Class I or II multiple gingival recession defects were treated with coronally advanced flap (CAF) procedures or CAF covering a material capable of supporting the flap, in a randomized controlled trial carried out by Bansal et al. [99]. This was a split-mouth study, and GR periodontal defects were randomly assigned to one of the treatment groups: test group (CAF + bioactive glass) or control group (CAF only). The analysis of clinical parameters (GR, CAL gain, keratinized tissue height) 6 months after surgery did not show significant differences in Class I/II GR defect regeneration between the groups. Both the treatments were effective to regenerate the gingival defects.

**Table 3:** Summary of clinical studies.

References	Materials	Number of Patients and Follow-Up
Humagain et al. [80]	PerioGlas^®^	16 patients 6 months
Keles et al. [81]	PerioGlas^®^	15 patients 6 months
Demir et al. [82]	Unigraft^®^ (200–420 μm)	29 patients 9 months
Cetinkaya et al. [83]	PerioGlas^®^	11 patients 60 months
Kaur et al. [84]	PerioGlas^®^	10 patients 6 months
Katuri et al. [85]	PerioGlas^®^, Grafton^®^	10 patients 12 months
Sculean et al. [86]	Enamel matrix protein derivative (EMD) and bioactive glass	10 patients 12months
Kumar et al. [87]	Bonelike^®^ (glass reinforced HA with α and β forms of tricalcium-phosphate)	10 patients 6 months
Satyanarayana et al. [88]	PerioGlas^®^	12 patients 12 months
Subbaiah and Thomas [89]	PerioGlas^®^	8 patients 3, 6, 9 months
Mistry et al. [90]	PerioGlas^®^	22 patients 6 months
Lysienko and Borysenko [91]	BG graft. BG doped with 1% silver and 0.5% copper	47 patients 6, 12 months
Slezák et al. [92]	NovaBone^®^	10 patients 3, 6 months
Grover et al. [93]	NovaBone^®^	12 patients 3, 6 months
Asmita et al. [94]	NovaBone Dental Putty^®^ graft or BG (PerioGlas^®^)	28 patients 6 months
Biswas et al. [95]	NovaBone Dental putty^®^	15 patients 3, 6, 9 months
Naqvi et al. [96]	NovaBone^®^	20 patients 6, 9 months
Saravanan et al. [97]	BG putty	20 patients 6, 9 months
Koduru et al. [98]	Nanocrystalline hydroxyapatite (Nc-HA) (Sybograf ™)	20 patients 3, 6, 9 months
Bansal et al. [99]	NovaBone^®^	10 patients 6 months

## 4. Discussion

### 4.1. In Vitro Tests

The purpose of this review was to investigate the progress made in the use of bioactive glass in regenerative periodontology over the past fifteen years. We found 24 documents that met the established inclusion criteria. In particular, we excluded all the works on microbiological aspects, because we wanted to focus the on the aspects of bone regeneration elicited by the bioactive glass alone or by the scaffolds/membranes containing it, in the treatment of periodontal disease. The BGs were tested against a wide variety of aerobic and anaerobic bacteria, showing an antibacterial effect without selecting for resistance, together with a good activity against biofilm formation [100]. The antibacterial effect seems due to the contact of BGs with biological fluids resulting in the increase of osmotic pressure and pH due to the leaching of ions from granules’ surface, thus making the surrounding environment hostile to microbial growth. However, the studies on the antibacterial properties of BGs are characterized by a wide heterogeneity linked to the bacterial species tested as well as the composition, size, concentration of the used BGs, and different methods to assess the antibacterial activity of different types of BGs [101]. Moreover, the importance of such a mechanism might be lessened in in vivo conditions, due to buffering of the system [102]. So, the antibacterial properties of BGs were improved by loading them with antibiotics or doping with bactericidal ions (such as silver) to avoid the emergence of resistant strains [13,16,52,91], but without clinical results of true significance in maxillary bones.

In recent years, in vitro studies have been mainly focused on: (1) the analysis of materials for the treatment of bone defects, (2) the addition of chemical elements to the bioactive glass already in use to enhance its osteo-regenerative effect, (3) the effect of new BG production techniques that could also influence its osteoinductive capacity, (4) the creation of new composites/membranes/scaffolds for guided tissue regeneration/bone regeneration.

We know that the periodontal area is histologically represented by four connective tissues, of which two are mineralized (cement and alveolar bone) and the other two are fibrous (lamina propria of the gingiva and periodontal ligament) [103]. In particular, the periodontal ligament is a peculiar connective tissue that contains at least three distinct populations of cells: fibroblasts, osteoblasts, or cementoblasts.

Regarding the cell types used in the cited papers, human and mouse cells were used, both of primary and immortalized origin.

The cells were placed in direct contact with the materials or with eluates/extracts of the material, to assess their cytocompatibility and/or cell attachment and/or cell proliferation and/or osteoinductive capacity.

Human periodontal ligament fibroblasts (hPDLF), taken from the patients for the study, were the most commonly used cells in in vitro studies for the evaluation of bioactive glass in periodontal bone regeneration. 

Fibroblasts of gingival origin, such as human gingival fibroblasts (hGF), have also been used in some in vitro studies.

In general, in our bibliographic research, cellular fibroblasts (deriving from the periodontal ligament or from the gingiva) were the most used to verify aspects of cytocompatibility and bone regeneration using bioactive glasses.

Human primary osteoblastic cells (POBs) derived from bone were also used. Such cells are widely utilized because they are easy to buy and do not require the approval of an ethics committee. POBs are isolated from the femoral trabecular bone tissue from the knee or hip joint region.

Other purchasable bone-derived cells are: (1) the human osteoblast (hOB) cell line, isolated from fetal or adult human bone, recognized as a model system for skeletal system studies, (2) immortalized cell lines such as the human osteosarcoma cell line (MG63), and (3) human primary osteogenic sarcoma (Saos-2 cells); the latter are widely used as osteoblastic cell models; (4) human dental pulp stem cells (hDPSC) isolated from human third molar teeth, which were also used to evaluate cytocompatibility and osteogenic differentiation.

In many studies cells of non-human origin, such as mouse or rat cells, were frequently used; in one paper we found that hamster cells were used.

The murine osteoblastic cell line (MC3T3-E1) was reported in various studies, being an osteoprogenetic line. Such line is widely used to evaluate (i) how the materials (e.g., BGs) act on the differentiation from osteoblasts into mature osteoblast phenotypes and (ii) osteogenic gene expression.

As cells included in the standard cytotoxicity tests (ISO 10933-5), the mouse fibroblasts (L-929) are immortalized cells derived from subcutaneous connective tissue, and only one paper between the one analyzed used them [69] to evaluate the cytotoxicity of materials’ extracts according to regulations.

Mouse dental papilla (MDPC-23) cells, a spontaneously immortalized cell line, are derived from fetal mouse molar papillae and are widely used in many in vitro studies for dentistry; such cells are referred as odontoblastic cell lines.

The in vitro studies found on murine cells were more specific; cell lines in the periodontal setting, cells of the parodontal ligament (rat periodontal ligament stem cells (pDLSCs)), and Wistar rats gingival fibroblasts (rGF) were used. Alternatively, dental but non-periodontal specific cells were used, such as rat pheochromocytoma PC-12 cells dental pulp cell line (KN-3), rats cementoblast (rC), rat primary osteoblastic (rPO) cells, rat osteoblasts calvaria (rOC).

Chinese hamster ovarian cells (CHO cell Line) were used for assessment of the cytotoxicity effects by Esfahanizadeh et al. [63].

With regard to the secondary outcomes, different authors found that, when human cells were used, BGs induced and/or increased cell viability and proliferation, and stimulated mineralized tissue formation [37,53,55,66,67,72,75], especially when BG nanoparticles were employed [55,66]. When fibroblast/osteocyte cell lines were utilized, cell viability, cell proliferation, cell differentiation were appreciated [60,64,70,71], in particular with mesoporous BG nanospheres/nanoparticles [54,68]. Some studies detected specific cell factors involved in the osteoinduction response [37,56,59], while BGs in membranes promoted healing [64,65,66,68,69]. Finally, antibacterial activity of BGs was demonstrated [52].

In general, the studies reported in this review to evaluate cytocompatibility and regenerative osteoinduction in vitro show heterogeneity in the cell types used and, more importantly, employ mostly non-periodontal and non-dental cells. The use of such a wide range of cells, of culture systems based on primary cultures or on immortalized lines, and different study designs makes it difficult to blend the results regarding the studies considered. Almost always, these studies performed tests aimed to analyze cell viability, cell proliferation, cell differentiation, and enhanced mineralized tissue formation that correspond to the selected secondary outcomes mentioned above. Nanostructured BGs and/or composites with BGs, in particular doped with boron, strontium, or niobium, seemed to guarantee better results considering the secondary outcomes. Zn or Ag could induce better in-vitro antibiofilm activity, with the important limits described above of their clinical significance. 

### 4.2. In Vivo Tests

The aim of this review was to investigate the advances made in the use of bioactive glass in periodontal defects. Considering this aim, few articles on in vivo studies were found in the period 2006–2021. Only seven articles related to our literature research were identified.

Different animal models were used in the studies: dogs, rabbits, mice, rats, with different follow-ups.

Of seven articles, three used the dog as an animal model, with a low number of animals.

Felipe et al. [77] implanted BG particles in six dogs, Lee et al. [78] utilized calcium phosphate glass in five dogs, and Carvalho et al. [76] investigated Perioglas^®^ and PRP in nine mixed-breed dogs.

Thus, different models of periodontal defects were analyzed in the three articles using the dog as an animal model, with different prognosis, different healing predictivity (e.g., 3-wall defects vs. critical size defects) and thus requiring different surgical strategies to be regenerated. Moreover, different healing times (60 days, 90 days) were used. On the contrary, the histological and histomorphometric analysis was the same in all the papers. 

Another animal model used in the in vivo studies to evaluate BGs in surgical therapy of the periodontal defect was the rabbit [59].

With regard to smaller animal models, the mouse/rat model was used. It is certainly a cheaper animal model, which allows an increase in the number of animals. These animal models compel to use a quite different dental context than the Primates’ one, or just an anatomical context different from the oral one, adding—to the difficult comparisons with the human periodontal disease model—the disturbing variable of the different anatomical site, where certainly there is no periodontal ligament. Therefore, in the few studies found the materials are implanted in different anatomical areas, calvaria in the mouse and calvaria, dorsal muscles, and mandible in the rat.

Granel et al. [71] showed that more than 30% of defect repair occurred after 90 days using BG-PCL scaffolds than the control sites left empty, with homogeneous mineralization and support bone remodeling.

Shah et al. [68] did not evaluate bone regeneration because no bone defect was created, but only a subcutaneous pocket; a membrane with BG was inserted in Wistar rats. The evaluation after one month only verified the good histocompatibility of the implanted material.

Fifteen wistar rats were used by Zhang et al. [79] as animal model for the in vivo study of a BG scaffold and a scaffold made of BG containing also strontium. Bilateral defects in the mandible were created and then filled with the biomaterial divided in three groups: non-treated control, BG scaffold, and strontium-BG scaffold. After 28 days from implantation the periodontal fenestration defects treated with strontium containing BG scaffolds showed greater new bone formation (46.67%) when compared to BG scaffolds (39.33%) and control unfilled samples (17.50%). The study showed that the use of strontium doped BG scaffolds decreased osteoclastogenesis, plus increased alveolar bone regeneration.

In these few articles found, the variability in the surgical procedure, in the follow-up times and in the different use of the BGs does not allow to properly compare the effect of BGs in the dog animal model. However, some considerations are possible. BGs have been demonstrated to achieve clearly better results than controls, especially with regard to bone regeneration. This can be ascribed also to the possibility of varying the composition of BGs and/or of producing composites. Strontium-doped BGs appear to guarantee better regenerative outcomes [79], as well as association with PRP or growth factors [59,76]. In particular, zinc seems to allow the control of the dissolution and replacement times of BGs in biological tissues; additionally, the release of zinc ions accelerates bone formation [104,105]. Another consideration concerns the use of membranes for GTR. Within the limitations of the studies available, it seems that the use of membranes in GTR both with particulate BGs and scaffolds was not of particular importance. This aspect significantly differentiates BGs from hydroxyapatite-based materials. Moreover, to envelope [59] or not [104] BGs by membranes did not influence the inflammatory outcome, that seemed to be feeble.

### 4.3. Clinical Studies

There are several topics regarding periodontal regenerative therapy [106]. However, considering exclusively the periodontal defect to be treated, the main issues are the surgical techniques, the materials used to treat the periodontal defects and the results over time [107,108,109,110,111,112]. The surgical strategy depends on the periodontal defect that has to be regenerated. The material used has to favor at least the regeneration of the periodontal ligament and of the alveolar bone. The stabilization of the results over time is considered in relation to the follow-up of the study design. This last parameter, which from a clinical point of view is the more valid the more extensive, has its rationale in relation to the chosen regenerative system. 

Despite a recently published definition regarding short- (6–12 months), medium- (13–59 months), and long-term (>5 years) periodontal surgery [112], undoubtedly most of the studies on the outcomes of periodontal regeneration procedures are concluded at 6 months or 1 year. The primary aims of the periodontal regenerative surgery consist in providing the clot mechanical stabilization in the regenerative space, allowing angiogenesis and avoiding the infection. The periodontal intrabony defects (which are considered in this review) could be self-maintaining space defects [113], i.e., intraosseous defects provided of a bone morphology fully defining the mechanical stabilization of the clot, or non-self-maintaining space defects. In last clinical cases, the degenerated tissue content, proper of the periodontal defect (periodontal pocket), is not removed (the provided surgical therapy is limited to the thorough removing of the only subgingival microbiota ecosystems); in alternative, if the pocket tissue is removed, a complementary system has to be provided to maintain the regenerative space; an exoskeleton or an endoskeleton system are possible choices [109]. The advantages of titanium reinforced PTFE membranes may therefore be related to space provision and to blood clot stabilization effects (exoskeleton system). Resorbable membranes do not have the same self-maintaining space characteristic and may be used alone only in contained defects. Non-contained defects treated with resorbable membranes may therefore benefit from the combined use of a grafting material acting as a scaffold (endoskeleton system) [86,109]. Mostly, the filling materials should not be grafted alone. The covering with membranes is needed, in particular if the graft consists in an alloplastic HA (e.g., demineralized bovine bone mineral—DBBM) [114].

Alternatively, grafts have to be combined with other compounds increasing their osteogenetic properties and stabilizing the graft shape against mechanical stresses (e.g., amelogenins, platelet-rich plasma, recombinant human platelet derived growth factor-BB, peptide P-15, etc.) [109,115]. Clinical outcomes of the use of bioactive agents when applied in addition to OFD, either alone or in association with grafts and/or barrier membranes, were evaluated. The studies concluded that there was evidence to support the use of amelogenins, either alone or in combination with grafts, to treat intraosseous defects effectively, and the additional use of a graft seemed to enhance the clinical outcome of amelogenins.

Deep intrabony defects with high self-maintaining space properties show the same regenerative clinical outcome by the only minimally invasive surgical techniques, as minimally invasive surgical technique [116] or single flap approach [117] without grafting or membrane covering (exoskeleton or an endoskeleton systems). Strictly speaking, the open flap debridement (OFD) surgical technique consists of quite a different surgical approach. It is considered a periodontal surgical procedure in which the supporting alveolar bone and root surfaces of teeth are exposed by incising the gingiva to provide increased access for scaling and root planing (the provided surgical therapy is limited to the thorough removing of the only subgingival microbiota ecosystems). While the efficacy of this treatment is debated, it is performed ancillary to any osseous resective or regenerative periodontal procedures. So, OFD is not a surgical approach specifically suitable for resective or regenerative procedures. Besides, a large flap, extended to the neighboring teeth and including also an eventual periosteal incision and/or vertical-releasing incisions, will be chosen in the presence of a severe and deep defect, involving three or four sides of the root, requiring ample visibility for instrumentation or/and the use of either endoskeletons and membranes (resorbable exoskeletons) or non-resorbable exoskeletons [109]. However, a wide series of specific surgical techniques are commonly identified as OFD.

Most of the clinical trials considered in this review, covering the last 15 years, are randomized controlled trials. Mostly, the papers considered patients suffering from chronic periodontitis and all patients were systemically healthy. The OFD was often the chosen surgical technique, and it was carried out with the removal of the tissue from the periodontal pocket. Considering clinically correct the chosen surgical technique through force of circumstance, all the studies considered highlighted a clinical advantage using BGs graft than the alone OFD along time, varying from 6 to 9 months [80,87,89,90]. In all the cases, the BG graft (endoskeleton) was not provided with a covering membrane and in one case it consisted of a BG reinforced HA with α and β forms of tricalcium-phosphate [81]. So, if the intraosseous defects were provided of self-maintaining space properties, the short-term follow-up was appropriate to demonstrate the healing acceleration effect (which is what can be detected with these study designs). Moreover, BG may not show the same disadvantage as alloplastic HA grafts, which would need to be combined or covered with membranes. On the other hand, if the OFD had been implemented with the removal of the pocket tissue in non-self-maintaining space defects, the favorable results obtained with the graft would derive most of all from a methodological bias.

The use of BG has been evaluated with different study designs, comparing or combining BG with compounds capable of generating periodontal regeneration (enamel matrix protein derivative—EMD, platelet pellet—PP, platelet-rich plasma—PRP) but without the mechanical properties to maintain the space of the periodontal defects. The studies showed a follow-up from 6 months to 5 years [81,82,83,84,86,95,96,97]. Mostly, the studies highlighted for both the grafted materials (BG vs. EMD/PP/PRP) the same regenerative properties. The regeneration showed stability both at short- and at medium-term. However, several studies found better short-term regenerative results using a BG putty combined with platelet rich fibrin (PRF) than PRF alone [96,97] or BG than PRF alone [95]. The different study designs could explain the results. However, the BG considered seem to achieve effective regenerative properties [92,93,94]. The comparison between HA/DFDBA and BG grafting as regenerative properties showed better clinical results with BG grafting than graft covered with bioresorbable membranes [90,91], both using OFD surgical technique. On the other hand, Koduru et al. [98] found similar clinical outcomes compared DFDBA with BG grafts, and Katuri et al. [85] obtained better results for the DFDBA grafts. However, both the study designs did not cover the grafted material with membranes and, besides, Katuri at al. [85] used a DFDBA combined in putty form. Within its inherent limitations, the present clinical review shows that BGs have effective properties of periodontal forming. Moreover, BGs could be advantageously grafted without being combined with other compounds or covered by membranes, as necessary for HA graft. This could achieve the periodontal surgical regeneration of the periodontal defect in a further minimally invasive way.

However, to summarize, it is difficult to determine which BG composition can allow the best clinical results or is more promising, because the clinical outcomes also depend on the specific clinical characteristics of periodontal defects, and therefore also on the chosen surgical therapeutic strategy. Moreover, a significant heterogeneity characterized the studies considered.

## 5. Conclusions

This review offers a literature overview of different applications of some of the most common BGs, and their in vitro, in vivo, and clinical results/outcomes in the surgical treatment of periodontal disease. 

The main topics on in vitro studies were on the analysis of cellular/molecular mechanisms induced by BGs on cells that could support tissue regeneration. As far as in vivo studies are concerned, the tissue/bone regeneration was considered on a few animal models.

The main focus was on their clinical use in regenerative surgery (periodontal soft and hard tissues) of periodontal intrabony defects.

Within the inherent limits of this review, mostly due to the diversified study designs, BGs show a wide ability to be tailored in relation to the aim of the experimental design or therapy. In the in vitro study, BGs demonstrated an excellent ability to promote osteogenic differentiation, cell proliferation, and viability. Despite all these interesting features, it is also important to mention that BGs have some drawbacks, namely low fracture resistance in load bearing applications and the tendency to crystallize upon thermal treatments [24].

The in vivo studies are scanty. However, BGs, in particular combined with PRP or natural polymers, seem to be able to induce tissue regeneration. The clinical outcomes by BGs are satisfactory. In particular, BGs seem to be an adequate alternative to HA/DFDBA as graft in periodontal regeneration in non-self-maintaining space defects. Furthermore, the possibility to have an ideal material that combine both endo- and exo-skeleton regenerative properties has been suggested.

As far as an in vitro, in vivo, and mostly clinical approaches are concerned, the considered studies were often not homogeneous in terms of BGs tested, experimental approach, in vivo and in vitro experimental model, clinical approach; in other words, as study design. The extended possibility of modifying BGs composition, of loading or doping or mixing with other materials, hindered comparisons among studies. Moreover, a suitable animal model is likely to provide more information than in vitro studies, in which tissue physiology and complex cellular interactions are neglected, even though animal experimental models present ample dissimilarities to human oral tissues and physiology [118]. Although clinical trials were mostly randomized and controlled, they were commonly carried out with a limited number of samples and limited follow-up over time. These constitute significant limitations to the study. Therefore, studies with a more significant sampling number would be needed, calculated on the basis of a specific, well-detailed experimental design. Currently, the main clinical need seems to consist in the development of BGs advantageously compared to HA/DFDBA in periodontal regenerative therapy, and more generally, oral regenerative surgery. Moreover, the development of feasible, effective, and advanced BG-based biomaterials with immunomodulatory capability for tissue regeneration represents a seductive scientific achievement.

## Data Availability

Not applicable.
